# Low-Cost Open Source Ultrasound-Sensing Based Navigational Support for the Visually Impaired

**DOI:** 10.3390/s19173783

**Published:** 2019-08-31

**Authors:** Aliaksei L. Petsiuk, Joshua M. Pearce

**Affiliations:** 1 Department of Electrical & Computer Engineering, Michigan Technological University, Houghton, MI 49931, USA; 2 Department of Material Science & Engineering, Michigan Technological University, Houghton, MI 49931, USA; 3 Department of Electronics and Nanoengineering, School of Electrical Engineering, Aalto University, Espoo FI-00076, Finland

**Keywords:** visually impaired, blind, assistive devices, sensory substitution, obstacle avoidance, sensors, ultrasonic sensing, ultrasound sensing, 3-D printing, additive manufacturing

## Abstract

Nineteen million Americans have significant vision loss. Over 70% of these are not employed full-time, and more than a quarter live below the poverty line. Globally, there are 36 million blind people, but less than half use white canes or more costly commercial sensory substitutions. The quality of life for visually impaired people is hampered by the resultant lack of independence. To help alleviate these challenges this study reports on the development of a low-cost, open-source ultrasound-based navigational support system in the form of a wearable bracelet to allow people with the lost vision to navigate, orient themselves in their surroundings and avoid obstacles when moving. The system can be largely made with digitally distributed manufacturing using low-cost 3-D printing/milling. It conveys point-distance information by utilizing the natural active sensing approach and modulates measurements into haptic feedback with various vibration patterns within the four-meter range. It does not require complex calibrations and training, consists of the small number of available and inexpensive components, and can be used as an independent addition to traditional tools. Sighted blindfolded participants successfully demonstrated the device for nine primary everyday navigation and guidance tasks including indoor and outdoor navigation and avoiding collisions with other pedestrians.

## 1. Introduction

According to the World Health Organization (WHO) [1,2], approximately 1.3 billion people live with some form of vision impairment, and 36 million of them are totally blind. The vast majority of the world’s blind population live in developing countries [3]. In addition, this challenge is falling on the elderly at an increasing rate, with the group of visually impaired people over 65 years of age growing with a per-decade increase of up to 2 million persons, which is faster than the overall population with visual impairments [3]. However, even in developed countries like the U.S. this is becoming an increasing problem because of several factors. First, the U.S. is aging: Americans 65 and older is projected to more than double from 46 million today to over 98 million by 2060, and their share of the total population will rise to nearly a quarter from 15% [4]. Second, the elderly in the U.S. are increasing financially vulnerable [5]. According to American Foundation for the Blind [6] and National Federation of the Blind [7], more than 19 million American adults between the ages of 18 and 64 report experiencing significant vision loss. For working age adults reporting significant vision loss, over 70% are not employed full-time, and 27.7% of non-institutionalized persons aged 21 to 64 years with a visual disability live below the poverty line [7].

Safe navigation and independent mobility are parts of everyday tasks for visually impaired people [8], and can only partially be resolved with the traditional white cane (or their alternatives such as guide canes or long canes). According to several studies [9,10,11], less than 50% of the blind population use white canes. For those that do use them, they work reasonably well for short distances as they allow users to detect obstacles from the ground to waist level [12]. Some blind people also use mouth clicks to implement human echolocation [13]. Echoes from mouth click sounds can provide information about surrounding features far beyond the reach of a white cane [14], but, unfortunately, not all visually-impaired people are able to use this technique.

Over the past few decades, several approaches have been developed to create sensory augmentation systems to improve the quality of life of people with visual impairments, which will be reviewed in the next section. It is clear developing a sensor augmentation or replacement of the white cane with a sensory substitution device can greatly enhance the safety and mobility of the population of people with lost vision [15]. In addition, there are sensory substitution products that have already been commercialized that surpass the abilities of conventional white canes. However, most of the commercially available sensory substitution products are not accessible to most people from developing countries as well as the poor in developed countries due to costs: (i) UltraCane ($807.35) [16], an ultrasonic-based assistive device with haptic feedback and the range of 1.5 to 4 meters; (ii) Miniguide Mobility Aid ($499.00) [17], a handheld device that uses ultrasonic echolocation to detect objects in front of a person in the range of 0.5 to 7 meters; (iii) LS&S 541035 Sunu Band Mobility Guide and Smart Watch ($373.75) [18] that uses sonar technology to provide haptic feedback regarding the user’s surroundings; (iv) BuzzClip Mobility Guide ($249.00) [19], a SONAR-based hinged clip which has three ranges of detection (1, 2, and 3 meters) and provides haptic feedback; (v) iGlasses Ultrasonic Mobility Aid ($99.95) [20] provides haptic feedback based on ultrasonic sensors with the range of up to 3 meters, (vi) Ray [21] complements the long white cane by detecting barriers up to 2.5 meters and announces them via acoustic signals or vibrations, (vii) SmartCane ($52.00 for India and $90.00 for other countries) [22,23] detects obstacles from knee to head height based on sonic waves and modulates the distance to barriers into intuitive vibratory patterns. It is thus clear that a low-cost sensor augmentation or replacement of the white cane with a sensory substitution device is needed. 

In the most recent comparative survey of sensory augmentation systems to improve the quality of life of people with visual impairments [24], assistive visual technologies are divided into three categories: (1) vision enhancement, (2) vision substitution, and (3) vision replacement. In addition, Elmannai et al. [24] provided a quantitative evaluation of wearable and portable assistive devices for the visually impaired population. Additional devices have been developed for white canes are based on ultrasonic distance measurements and haptic [25,26] or audio [27] feedback. Amedi et al. [28] introduced an electronic travel aid with the tactile and audio output, that uses multiple infrared sensors. Bharambe et al. [29] developed a sensory substitution system with two ultrasonic sensors and three vibration motors in form of a hand device. Yi et al. [30] developed an ultrasonic-based cane system with haptic feedback and guidance in audio format. Pereira et al. [12] proposed a wearable jacket as a body area network and Aymaz and Çavdar [31] introduced an assistive headset for obstacle detection based on ultrasonic distance measurements. Agarwal et al. [32] developed an ultrasonic smart glasses. De Alwis and Samarawickrama [33] proposed a low-cost smart walking stick which integrates water and ultrasound sensors.

In addition to the devices based on the use of acoustic waves, there are also a number of projects which utilize more complex systems based on computer vision [34,35,36,37], machine learning technique [38], and GPS/GSM technologies [39] and provide information regarding the navigation and ambient conditions. 

Tudor et al. [40] designed an ultrasound-based system with vibration feedback, which could be considered as the closest prototype to the device developed and tested here. The ultrasonic electronic system [40] utilizes two separate ultrasound sensors for near (2 to 40 cm) and far (40 to 180 cm) distance ranges respectively. Tudor et al. [40] use simple linear pulse width modulation dependency for both vibration motors. Most of these projects allow to navigate within the distance range of 4 m, but suffer from drawbacks related to cost and complexity and thus accessibility to the world’s population of visually impaired poor people. One approach recently gaining acceptance for lowering the costs of hardware-based products is the combination of open source development [41,42,43] with distributed digital manufacturing technologies [44,45]. This is clearly seen in the development of the open source self-replicating rapid prototyper (RepRap) 3-D printer project [46,47,48], which radically reduced the cost of additive manufacturing (AM) machines [49] as well as products that can be manufactured using them [50,51,52] including scientific tools [42,53,54,55,56,57], consumer goods [58,59,60,61,62,63], and adaptive aids [64]. In general, these economic savings are greater for the higher percentage of the components able to be 3-D printed [65,66].

In this study, a low-cost, open-source navigational support system using ultrasonic sensors is developed. It utilizes one ultrasound sensor with one or two vibration motors (depend on the designed model) in a 3-D printed case to make the system compact and easily fixed on the wrist as a bracelet. The developed Arduino software performs distance measurements in the full sensor range from 2 cm to 4 m according to the ultrasound sensor specifications and implements an algorithm that uses the motors as low- and high-frequency vibration sources. The distance modulation technique consists of splitting the entire four-meter measurement range into four bands as described in Section 2.4, and of implementing the non-linear duty cycle modulation for the most significant middle band of 35 cm to 150 cm. The system can be largely digitally manufactured including both the electronics and mechanical parts with conventional low-cost RepRap-class PCB milling [67] and 3-D printing. The system is quantified for range and accuracy to help visually-impaired people in distance measurement and obstacle avoidance including the minimal size of the object. According to [24,68], the proposed system partially fulfills the Electronic Travel Aid (ETA) requirements, which consist of providing tactile and/or audible feedback on environmental information that is not available using traditional means such as white cane, guide dog, etc. Sighted blindfolded participants tested the device for primary everyday navigation and guidance tasks including: (a) walk along the corridor with an unknown obstacle, (b) bypass several corners indoors, (c) walk through the staircase, (d) wall following, (e) detect the open door, (f) detect an obstacle on the street, (g) bypass an obstacle on the street, (h) avoid collisions with pedestrians, (i) interact with known objects.

## 2. Materials and Methods

### 2.1. Design

**Figure 1:**
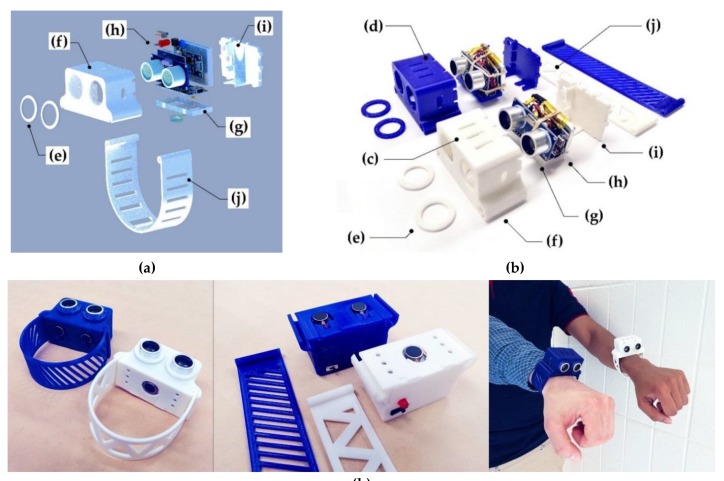
Parts of an open-source navigational support with 3-D printable case components: (**a**) 3-D prototype; (**b**) Components; (**c**) Model 1 with one vibration motor; (**d**) Model 2 with two vibration motors; (**e**) Locking rings; (**f**) Case; (**g**) Vibration pad; (**h**) Sensor core; (**i**) Back cap; (**j**) Bracelet; (**k**) Assembly.

An open-source navigational support with the 3-D printable case components was developed in form of two models of wearable bracelet with one and two vibration motors respectively (Figure 1) to help visually impaired people in distance measurement and obstacle avoidance. The system is based on a 5-volt HC-SR04 ultrasonic sensor [69], which uses SONAR (originally an acronym for sound navigation ranging) to determine the distance to an object in the range of 0.02–4 m with a measuring angle of 15 degrees. It detects obstacles in front of the user’s body from the ground to the head and above, and provides haptic feedback using a 10 mm flat vibration motor [70], which generates oscillations with variable frequency and amplitude depending on the distance to the obstacle. The microcontroller unit allows us to coordinate the operation of the motor and the sensor based on the developed algorithm, uploaded as a C-code to Arduino.

The device can be placed on the right or left hand, and it does not prevent the use of the hand for other tasks. It conveys point-distance information and could be used as a part of an assembly of assistive devices or as an augmentation to a regular white cane. In that way, the active sensing approach [15] was utilized, in which a person constantly scans the ambient environment. This method allows a user to achieve better spatial perception and accuracy [15] due to the similarity to natural sensory processes [71,72]. 

### 2.2. Bill of Materials

The system was prototyped for people with no engineering skills and the lack of available materials, so they can finish the assembly with the minimal toolkit. The bill of materials is summarized in Table 1. A 5V DC-DC boost step-up module can be considered as an optional component that can be used for battery life extension.

### 2.3. Assembly

**Figure 2:**
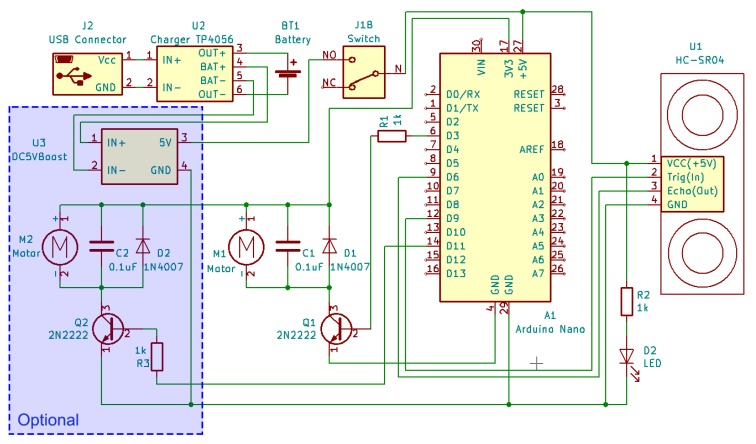
Electrical circuit.

After 3-D printing all the necessary components, electronic parts should be soldered together following Figure 2 and assembled to a sensor core with the vibration motor.

The Arduino Nano board should be programmed with the code available at [73], and the electronic core assembly should be placed in the 3-D printed case to finish the whole assembly (Figure 1k). All the CAD models and STL files are available online under an open-source CC BY-SA 3.0 license (Creative Commons – Attribution – Share Alike) [74]. The hand bracelet (Figure 1j) has an online option for customization [75], so a person with no experience with complicated 3-D modeling software could print the part after adjusting it to their hand size. For Arduino programming, it is necessary to download free open source Arduino IDE [76]. The final assembly of the sensor core and 3-D printed case components is demonstrated in the supplementary material (Video S1).

### 2.4. Operational Principles

The ultrasonic sensor emits acoustic waves at the frequency of 40 kHz, which travel through the air and reflects from objects within the working zone. Every measurement cycle it sends a 10 µs trigger input pulse and an 8-cycle sonic burst at the speed of sound and its reflection from an object is received by an echo sounder [69]. It receives a 150 µs – 25 ms (38 ms in case of no obstacle) output echo pulse, T_echo_, which width linearly depends on the distance to the detected obstacle:

T_echo_ / k = D, (1)

where T_echo_ is the output echo pulse width [µs], D is the measured distance to an obstacle [cm], k = 58 (µs/cm) is the conversion factor given by the sensor datasheet.

The measurement cycle, T_measure_, specified in the Arduino program and represents a constant time duration declared by a developer, but according to the sensor datasheet, this time duration should be no less than 60 ms [69]. The distance to the object is measured by the time delay between sending and receiving sonic impulses in the Arduino program.

A single exponential filter [77] was used to smooth noisy sensor measurements. It processes the signal with the desired smoothing factor without using a significant amount of memory. Every time a new measured value y_t_ is provided, the exponential filter updates a smoothed observation, S_t_:

S_t_ = α · y_t_ + (1−α) · S_t-1_, 0 < α < 1, (2)

where S_t−1_ – is the previous output value of the filter, y_t_ is a new measured value, α = 0.5 is the smoothing constant. 

**Figure 3:**
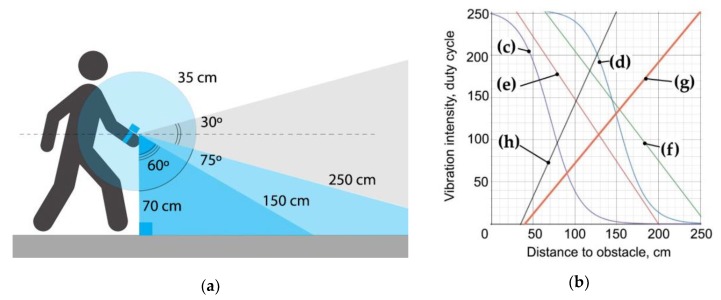
The ultrasonic sensor operating principles: (**a**) The principal distances (not to scale); (**b**) Calibration of the optimal duty cycle equation for the distance range of 35 cm to 150 cm, where (**c**) M_DC_ = 127 + 127 · tanh (−(D − 70) / 35); (**d**) M_DC_ = 127 + 127 · tanh (-(D - 150) / 35); (**e**) M_DC_ = 296 – 1.5 · D; (**f**) M_DC_ = 335 – 1.3 · D; (**g**) M_DC_ = −77 + 2.2 · D; (**h**) M_DC_ = −48 + 1.2 · D.

Total measurement time consists of the traveling time caused by the finite speed of sound and the delay necessary for measurements. The time delay caused by the finite speed of sound, T_max travel_, is:

T_max travel_ = D_max_ / V_sound_ = 2 · 4 / 340 = 8 / 340 = 24 (ms), (3)

where, D_max_ is the maximum measured distance to an obstacle and V_sound_ is the speed of sound in air. The measured distance is modulated with vibration amplitude and translated in real-time as a duty cycle parameter from the Arduino board (Figure 3). Distances up to 35 cm are characterized by single vibration pulses with a relatively high periodicity. Distances from 150 to 250 cm are characterized by single pulses with low periodicity, and distances above 250 cm are modulated with two-pulse beats.

**Figure 4:**
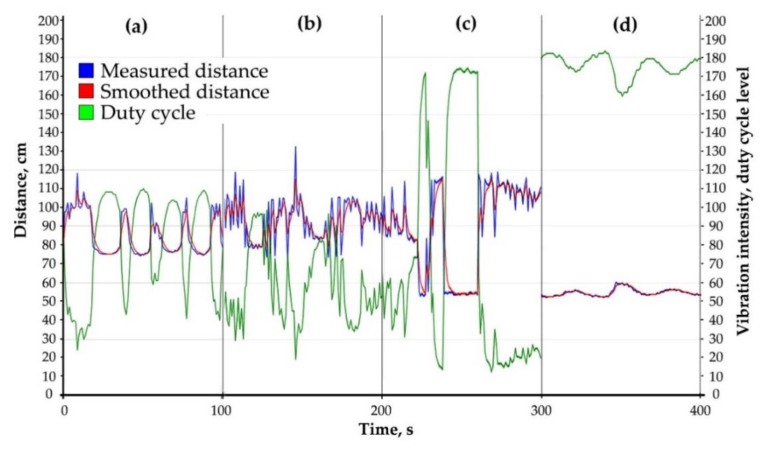
Calibration procedure of the duty cycle modulation based on hyperbolic tangent function (4): (**a**) Hand swinging; (**b**) Wall following; (**c**) Obstacle detection; and (**d**) Curbs tracking.

An optimal duty cycle equation (Figure 3c) for the most common distance range of 35–150 cm was found during experiments and calibrations (Figure 3b). The generated duty cycle for the Arduino output, M_DC_ is:

M_DC_ = m + m · tanh (–(D–k) / b) = 127 + 127 · tanh(– (D – 70)/35), 0 < M_DC_ < 255, (4)

where m = 127, k = 70 and b = 35 are the calibrated parameters, and D is the measured distance in the range of 35 cm to 150 cm. This modulation law is based on hyperbolic tangent function, tanh, (Figure 3c), which is close to the inverse of pain sensitization in its shape [78,79] and demonstrated the best efficiency in most common tasks (Figure 4).

**Figure 5:**
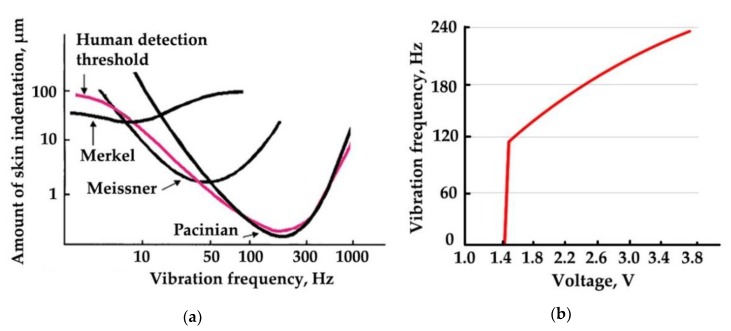
Human haptic sensitivity and vibration motor characteristics: (**a**) Psychophysically determined thresholds for detection of different frequencies of vibrotactile stimulation (adapted from [82]); (**b**) Vibration motor performance (adapted from [70]).

According to [80,81], there are four major types of tactile mechanoreceptors in human skin: (1) Merkel’s disks, (2) Meissner’s corpuscles, (3) Ruffini endings, and (4) Pacinian corpuscles. Meissner’s corpuscles respond to high amplitude incentives with low frequency and Pacinian corpuscles, in turn, respond to low amplitude incentives with high frequency. Thus, varying amplitude and frequency of vibrations, it is possible to activate these mechanoreceptors separately, which increases the working range of sensitivity levels (Figure 5a). The vibration pad (Figure 1g) with the vibration motor are in contact with the skin on the outer area of the wrist. As the nominal frequency characteristics of the motor (Figure 5b) do not cover the full range of human threshold for detection of vibrotactile stimulation, switching the motor on and off at different intervals allows us to simulate low-frequency pulsations.

Estimated current for the whole device is at the level of 50 mA assuming that the vibration motor works 40% of the time. According to this, a 400 mAh battery will provide us with 8 hours of autonomous work, which is an efficient amount of time for test purposes as well as for general use if a blind person was walking throughout the entire working day.

Finally, the cost saving in percent (P) of the device was determined by:

P = (c – m) / c · 100,(5)

where c is the commercial cost of an equivalent device and m is the cost in materials to fabricate the open source device. All economic values are given in U.S. dollars.

### 2.5. Testing of the Device

Since there are no well-established tests for sensory substitution devices, the experimental setup was based on previous experience. García et al. [26] conducted an experiment with eight blind volunteers and evaluate the results in form of quiz, where participants noted the efficiency in obstacle detection above the waistline. Pereira et al. [12] evaluated their prototype on both blind and sighted participants in five different scenarios to simulate the real-world conditions, including head-, chest-, foot-level obstacles, and stairs. Maidenbaum et al. [15] performed a set of three experiments with 43 participants (38 of them are sighted blindfolded) to evaluate their prototype on basic everyday tasks, including distance estimation, navigation, and obstacle detection. Nau et al. [83] proposed an indoor, portable, standardized course for assessment of visual function that can be used to evaluate obstacle avoidance among people with low or artificial vision.

Summarizing the experience of previous researchers, the set of experiments used to test the devices here consists of indoor and outdoor, structural and natural environment in order to explore the intuitiveness of the developed device and its capabilities in everyday human tasks.

Five sighted blindfolded lab researchers took part in a series of tests, the main purpose of which was to collect the necessary information about adaptation pace, usability, and performance of the developed system. The experiments were conducted in a familiar indoor and outdoor environment for the users. 

**Figure 6:**
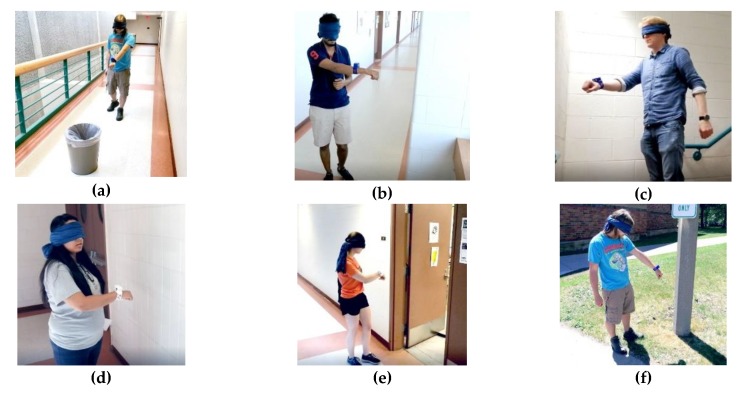

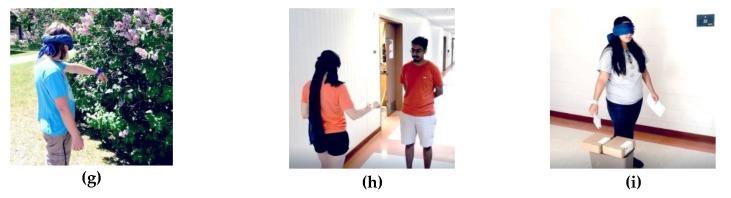
Testing procedure. (**a**) Walk along the corridor with an unknown obstacle; (**b**) Bypass several corners indoors; (**c**) Walk through the staircase; (**d**) Wall fallowing; (**e**) Detect the open door; (**f**) Detect an obstacle on the street; (**g**) Bypass an obstacle on the street; (**h**) Avoid collisions with pedestrians; (**i**) Interact with known objects.

Participants were assigned to the following nine tests (Figure 6):

(a) Walk along the corridor with an unknown obstacle
(b) Bypass several corners indoors
(c) Navigate a staircase
(d) Wall following
(e) Detect the open door
(f) Detect an obstacle (shrub plant/road sign) outdoors
(g) Bypass an obstacle outdoors
(h) Avoid collisions with pedestrians
(i) Interact with known objects (bin/cardboard boxes)

Each participant had a 10-minute training before nine tests with two attempts, one attempt using the Model 1 (the white device, Figure 1c) and one attempt using the Model 2 (the blue device, Figure 1d). The tasks were to complete a distance of approximately 10 meters (20 steps) with obstacles (pedestrian, road sign, etc.) or with landmarks (wall, door, steps, etc.). Success was counted if the participant has finished the path relying only on the sensing device. Any collision with an obstacle or difficulty in passing the test path was considered a failure. The time spent on passing the test was not taken into account. Results of the experiments presented in Table 2. Some of the experiments were recorded and included in the supplementary material (Video S1).

## 3. Results and Discussion

All versions of the device were built for less than $24 USD each in readily available purchased components and 3-D printed parts. The economic savings over generally inferior commercial products ranged from 80.2–97.8% for the base system to 73.5–97.0% for the optional module system.

The devices were tested to demonstrate that it has intuitive haptic feedback as outlined above. The device range and accuracy was found to allow a person with a lost eyesight to detect objects with the area of 0.5 m^2^ or more within the distance range of up to 4 m. To omit head-level obstacles the user must sweep the head area along the direction they are moving. During the experiments was also found that it is possible to track objects with the moving speed of up to 0.5 m/s within the one-meter distance range.

As can be seen from Table 2, the failure rate is 6.7%, with the majority of failures occurring in experiments with Model 2 (the blue device with two vibration motors, Figure 1d). Some participants experienced difficulty navigating a staircase, as well as finding and avoiding unfamiliar obstacles indoors and outdoors.

The preliminary testing of the device was determined to be a success based on the majority of the participants being able to complete the nine tasks outlined in the methods section. All participants during the experiments noted the effectiveness of the haptic interface, the intuitiveness of learning and adaptation processes, and the usability of the device. The system produces fast response and allows a person to detect objects that are moving. It naturally complements primary sensory perception of a person and allows one to detect moving and static objects.

The system has several limitations. First, for the developed system, it is necessary to note a narrow scanning angle and a limited response rate, which is expressed in ignoring the danger posed by small and fast-moving objects. Second, the low spatial resolution of the system is also noted. Thus, in the conditions of an outdoor street environment, it was difficult for the experiment participants to track road curbs and determine the change in the level of the road surface. Third, indoors, soft fabrics, such as furniture and soft curtains, as well as indoor plants, can cause problems with distance estimation caused by acoustic wave absorption. In open outdoor areas, determining the distance can be difficult on lawns with high grass and areas with sand. In addition, given the increase in the threshold of sensitivity with age [84], the performed experiments do not cover the diversity of the entire population of people with visual impairments.

This is a preliminary study on the technical specifications of the new device and a much more complete study is needed on human subjects including blind subjects. Future work is needed for further behavioral experimentation to improve data acquisition methods, obtain more data, and perform a comprehensive statistical analysis of the developed system performance. This will allow designers to utilize achievements in haptic technologies [85] and to improve the efficiency of its tactile feedback, since the alternation of patterns of high-frequency vibrations, low-frequency impulses and beats of different periodicity can significantly expand the range of sensory perception. Similarly, improved sensors could expand range and improved electronics could increase the speed at which objects could be detected. Minor improvements can also be made to the mechanical design to further reduce the size, alter the detector angle to allow for more natural hand movement, and improved customizable design to allow for individual comfort settings as well as aesthetics.

## 4. Conclusions

The developed low-cost (<$24 USD), open-source navigational support system allows people with lost vision to solve the primary tasks of navigation, orientation, and obstacle detection (>0.5 m^2^ stationary within the distance range of up to 4 m and moving up to 0.5 m/s within the distance range of up to 1 m) to ensure their safety and mobility. The devices demonstrated intuitive haptic feedback, which becomes easier to use with short practice. It can be largely digitally manufactured as an independent device or as a complementary part to the available means of sensory augmentation (e.g., a white cane). The device operates in similar distance ranges as most of the observed commercial products, and it can be replicated by a person without high technical qualification. Since the prices for available commercial products vary from $90–800 USD, the cost savings ranged from a minimum of 73.5% to over 97%.

## Figures and Tables

**Table 1:** Bill of materials for the open-source ultrasound-based navigational support.

Component	Quantity	Cost, USD
3-D printed case	1	0.65
3-D printed back cap	1	0.25
3-D printed bracelet	1	0.40
3-D printed vibration motor pad	1	0.05
3-D printed locking rings	2	0.05
Arduino Nano	1	3.80
Ultrasonic Sensor HC-SR04	1	1.83
Flat 10 mm 3 V vibration motor	1	1.40
400 mAh lithium polymer battery	1	7.49
Micro USB 5 V 1 A 18650 TP4056 lithium battery charger	1	1.20
*DC-DC 5V boost step-up module (optional)	*1	*5.99
Slide switch	1	0.40
0.25W 1 kΩ resistor	2	<0.01
Ceramic 0.1uF capacitor	1	0.07
1N4007 diode	1	0.08
2N2222 transistor	1	0.07
5 mm LED	1	0.07
**Total cost, USD**	17.82 *(23.81 with optional module)	

**Table 2:** Results of the experiments.

Participants	Tests																	
	a		b		c		d		e		f		g		h		i	
	Device Model																	
	1	2	1	2	1	2	1	2	1	2	1	2	1	2	1	2	1	2
1	•	‒	•	•	•	•	•	•	•	•	•	•	•	•	•	•	•	•
2	•	•	•	•	•	•	•	•	•	•	•	•	•	•	•	•	•	•
3	•	•	•	•	•	•	•	•	•	•	•	•	•	•	•	•	•	‒
4	•	•	•	•	‒	‒	•	•	•	•	•	•	•	‒	•	•	•	•
5	•	•	•	•	•	•	•	•	•	•	•	•	•	‒	•	•	•	•

• Success, ‒ Failure.

## Supplementary Materials

The following are available online at https://youtu.be/FA9r2Y27qvY. Video S1: Low-cost open source ultrasound-sensing based navigational support for visually impaired.
